# Implementation barriers, facilitators, and fidelity of a mobile money-based savings tool for pregnant women in the Analamanga region of Madagascar: an ex-post process evaluation

**DOI:** 10.1186/s43058-026-01055-1

**Published:** 2026-08-11

**Authors:** Lisa Bogler, Sebastian Vollmer, Zavaniarivo Rampanjato, Harizaka Emmanuel Andriamasy, Bítia Vieira, Louis Noël Schäfer, Elsa Rajemison, Onja Gabrielle Ravololohanitra, Andriamampianina Ralisimalala, Till Bärnighausen, Samuel Knauss, Julius Valentin Emmrich

**Affiliations:** 1 Centre for Modern Indian Studies and Department of Economics, University of Goettingen, Göttingen, Germany; 2 Global Digital Last Mile Health Research Lab, Charité Center for Global Health, Charité - Universitätsmedizin Berlin, Berlin, Germany; 3 Heidelberg Institute of Global Health, Medical Faculty and University Hospital, University of Heidelberg, Heidelberg, Germany; 4 Ministry of Public Health of the Republic of Madagascar, Antananarivo, Madagascar; 5 Doctors for Madagascar, Antananarivo, Madagascar; 6 Centre d‘études et de recherche en économie de la santé (CERES), Faculté d’économie, de gestion et de sociologie, Université d’Antananarivo, Antananarivo, Madagascar; 7 Department of Global Health and Population, Harvard T.H. Chan School of Public Health, Boston, Massachusetts USA; 8 Africa Health Research Institute (AHRI), Mtubatuba, KwaZulu-Natal South Africa

**Keywords:** mHealth, Health financing, Maternal health, Health wallet, Mobile money, Madagascar, Implementation fidelity, Process evaluation

## Abstract

**Background:**

Mobile payment services could improve access to obstetric care by easing saving, raising revenue, and pooling resources. Between 2019 and 2022, a mobile money-based savings tool for obstetric care was implemented in the Analamanga region of Madagascar following a randomized controlled trial design. We conducted an ex-post process evaluation of the intervention.

**Methods:**

We assessed implementation fidelity and reach of the trial using five indicators from a population-based survey of 6,234 women who were pregnant during the intervention period and lived in catchment areas of 63 public primary-care health facilities (Centres de Santé de Base, CSBs) included in the trial: whether a woman (a) had heard of the tool, (b) could explain what it was, (c) had registered with it, (d) had used it to save, (e) had used it to pay for costs of childbirth. Combining the population-based survey with a healthcare provider survey and in-depth interviews with women, healthcare workers, and implementation staff, we examined factors associated with implementation quality, and barriers and facilitators to take-up.

**Results:**

Sensitization led to statistically significantly higher take-up by women living in catchment areas of intervention CSBs compared to control CSBs: 39% vs 13% of surveyed women had heard of the tool, 33% vs 9% knew what it was, 15% vs 4% had registered, 15% vs 3% had saved with it and 10% vs 2% had paid with it. However, awareness and registration varied across intervention CSBs (4%-78%, 0%-54%). Key barriers to take-up included technical difficulties faced by providers, a disruption of informal benefits for providers, and mobile phone access. Learning about the tool from midwives was a facilitator.

**Conclusion:**

This study highlights the complexity of introducing a mobile money-based savings tool for obstetric care and the importance of evaluating implementation fidelity to understand trial impact. Strengthening community mobilization, providing more personalized support, and improving intervention compatibility with routine workflows could improve intervention delivery. User-centred design and phased roll-out allowing for continuous improvement cycles may help optimize effective implementation at scale.

**Trial registration:**

The 4MOTHERS trial was registered on March 12, 2021, in the German Clinical Trials Register (DRKS), identifier: DRKS00014928.

Contributions to the literature

- Demonstrates how provider-side disruption of informal payment benefits can undermine digital health financing tools.
- Provides empirical evidence on facility-level variation in implementation fidelity for a mobile health wallet.
- Identifies midwives as critical trusted intermediaries.
- Identifies misalignment between intervention design and routine clinical workflows as key barrier to implementation in low-resource maternal care settings.

## Background

Access to obstetric care remains restricted in low-resource settings in sub-Saharan Africa, with low household income suggested as a major barrier [1], especially where most health care expenses are paid out-of-pocket. Saving could mitigate the financial strain, but is especially difficult for poorer, disadvantaged households [2]. Saving financial resources with mobile payment services, i.e. in a mobile money account, means that they are not immediately at hand for sudden demands and therefore more protected than savings in form of cash. Mobile money could therefore provide a technology to ease saving and to pay for care.

With 545 maternal deaths per 100,000 live births in sub-Saharan Africa in 2020 and most deaths being preventable [3], a savings tool for pregnancy and childbirth could save lives by improving access to formal obstetric care, particularly by improving affordability and promoting health seeking behavior. Furthermore, the peripartum period could be a suitable period of time for using a purpose-specific savings tool as the predictable timing and costs of childbirth create a clear savings goal.

With the rise in mobile phone subscriptions [4], mobile money has become an important tool for financial transactions across sub-Saharan Africa [5]. Evidence suggests that mobile money users save more than non-users [6, 7], although the causal link is not fully established [8].

The use of mobile money-based services is also associated with better healthcare behavior: Evidence from Kenya suggests that users of mobile money services are more likely to seek formal care after a health shock than non-users [9]. Another study finds that pregnant women in households using mobile money were more likely to take up recommended antenatal care in Uganda [10]. Similar findings come from rural Ghana, where mobile money adoption seems correlated with enhanced healthcare utilization [11]. While some of these studies suggest increased borrowing as a possible pathway [9, 12], the impact through savings and the use of savings-focused mobile money tools remain less explored.

Despite the proliferation of digital health financing interventions, evidence on implementation processes is limited. Most studies focus on utilization, coverage, and outcomes only, without opening the “black box”, i.e. without assessing what influences utilization, coverage, and outcomes. Process evaluations are needed to understand how and why or why not complex interventions work [13].

The non-governmental organization Doctors for Madagascar developed the Mobile Maternal Health Wallet (MMHW), a mobile money-based savings tool to help pregnant women mitigate out-of-pocket expenditures for obstetric care by allowing users to save and pay for healthcare services. Prior work identified a high acceptability and perceived usefulness of the MMHW to improve access to healthcare among Malagasy women [14]. We implemented the 4MOTHERS trial, a randomized controlled trial in the Analamanga region of Madagascar to evaluate its impact. The impact evaluation of the trial, presented elsewhere, reported variation in implementation fidelity and did not find a statistically significant impact on maternal and neonatal health outcomes [15]. A second study on the same trial conducted a mixed methods doer/non-doer analysis to examine what personal factors and household decision-making processes influenced enrolment decisions concerning the MMHW among women who knew about it [16].

In this study, we present an ex-post process evaluation of the trial to assess the fidelity and reach of the MMHW intervention in greater detail. Using the population-based survey and the survey among healthcare providers collected for the impact evaluation, we analyze individual-level and provider-level factors associated with implementation fidelity. We further use results from in-depth interviews with women, healthcare workers, and implementation team members to enrich the quantitative data. While the impact paper [15] reported take-up rates as part of describing trial conditions, this study uses these indicators to systematically analyze variation in implementation fidelity across facilities. While Schäfer et al. [16] examined individual-level enrollment decision among women aware of the tool, this study adds provider-level perceptions, facility-level characteristics, and implementation team perspectives. The objective is to understand the observed variation in implementation fidelity and identify barriers and facilitators from the perspectives of women targeted as beneficiaries and healthcare providers.

## Methods

### Study design

The evaluation of the MMHW was designed as a hybrid effectiveness-implementation trial, including a randomized controlled trial to evaluate the impact of the MMHW as well as a process evaluation using a mixed-methods approach [17]. This study contains the results of the process evaluation and is primarily based on quantitative data, while findings from qualitative in-depth interviews, which had been conducted with a different focus, were used to enrich the quantitative analysis. Quantitative surveys and qualitative in-depth interviews occurred in parallel. With the quantitative data, we descriptively assessed pregnant women’s engagement with the MMHW, i.e. whether they had heard of, registered with or had used the MMHW, created a measure of low and high implementation quality, and analyzed individual-level and provider-level factors associated with implementation quality. The qualitative information was used to enrich the interpretation and discussion of quantitative results.

### Study setting and design of the randomized controlled trial

The MMHW intervention was implemented in three districts of the Analamanga region in Madagascar (Antananarivo Renivohitra, Atsimondrano, Avaradrano) by the non-governmental organization Doctors for Madagascar. Study design and intervention, including a map of the implementation area, are described in a published study protocol [17]. All public-sector primary-care health facilities (Centre de Santé de Base, CSB) in the study region that performed antenatal care (ANC) were eligible for inclusion. The 63 eligible CSBs were randomized into intervention and control groups. Randomization was stratified based on the number of ANC visits in 2017 and the capacity to perform deliveries, creating 6 strata (<1750 ANC visits/year and delivery services, <1750 ANC visits/year and no delivery services, 1750–3500 ANC visits/year and delivery services, 1750–3500 ANC visits/year and no delivery services, >3500 ANC/year and delivery services, >3500 ANC visits/year and no delivery services). The control group included 32, the intervention group 31 CSBs. Additionally, four public referral hospitals for maternal care in the Analamanga region, as defined by the Malagasy Ministry of Health, were included in the intervention group. All intervention CSBs received the intervention package by May 2020, with registration open and sensitization activities ongoing until December 2022.

### Description of intervention

The MMHW package had two main components. The first was targeted at pregnant women attending antenatal care (ANC) visits or giving birth at healthcare facilities. Women who registered with the MMHW received a SIM card to save in the MMHW and to pay with these savings at the intervention CSBs and referral hospitals. Deposits could be made anytime during pregnancy and up to 40 days postpartum and contributions from relatives or others could be received on their account. Women did not have to own a mobile phone to participate as the SIM card could be used with any available phone. Registered users received additional benefits: Costs for preventive drugs received during ANC visits (iron and folic acid tablets (on average 1800 MGA or 0.46 USD, maximum 3000 MGA or 0.77 USD^1^), Mebendazole (on average 200 MGA or 0.05 USD, maximum 500 MGA or 0.13 USD^1^)) and for ambulance transfer to referral hospitals for obstetric and neonatal emergencies (on average 180,000 MGA or 45.9 USD, maximum 300,000 MGA or 76.5 USD^1^) were covered. Doctors for Madagascar offered a 50% matching of deposits made to the MMHW if savings were used to pay for maternal health services. Additionally, users could receive up to two free obstetrical ultrasound examinations.

The second component was directed at public primary healthcare facilities. These facilities received a one-time training on using the MMHW and repeated capacity-building and refresher courses around maternal and neonatal health for health workers in maternity wards and ANC departments, led by trainers of the Ministry of Health. Financial incentives were also provided. For each birth by a beneficiary, the healthcare professional in charge of the birth received 10,000 MGA (2.44 USD^1^). The pharmacists or other staff responsible for taking photo evidence of claims received 2,000 MGA (0.51 USD^1^) per patient and month for each claim sent via the MMHW platform^2^. Community health workers received 1,000 MGA (0.24 USD^1^) for each woman referred to a facility for ANC and included in the program. Additionally, medical equipment, including blood pressure monitors, stethoscopes, examination beds, bedpans, and baby scales, was distributed to maternity units and community health workers received resources (backpack, t-shirt, cap, waistcoat) at partner CSBs.

### Intervention roll-out

The intervention roll-out started in November 2018, with payments through the MMHW possible until December 2022. CSBs in the intervention group received the training on the use of the MMHW between January 2019 and May 2020. Sensitization campaigns to encourage registration with the MMHW continued in intervention CSBs and their catchment areas until April 2022, using different strategies. Firstly, community health workers identified newly pregnant women in their community and conducted home visits to encourage them to go to the CSB for antenatal consultations and to inform them about the MMHW. Per month, a minimum of 25 such home visits per CSB catchment area were targeted, but this varied between 0 during the COVID-19 pandemic and 55 at other times and depended on the number of newly pregnant women in the area. Secondly, women visiting participating health facilities for ANC were informed about the MMHW during their visit. Each CSB had set dates for first antenatal consultations and on each such date, the MMHW project team held short information sessions in the waiting room or courtyard of the CSBs, followed by home visits for registration. Former beneficiaries were also mobilized about once every three months in each CSB to meet with small groups of 4 to 6 pregnant women, an activity called “réunion de proximité”, to share their experience with the MMHW and convince hesitant women to register. The MMHW project team used these meetings to understand rumors and collect problems with the MMHW in order to solve them and address them proactively in future sensitization events. Lastly, community-wide awareness-raising sensitization events were organized quarterly with artists in 2019. Between 30 and 80 women registered with the MMHW per event. Between 2020 and 2022, 42 of the most effective community health workers were selected and, together with MMHW agents, used megaphones to spread the word while walking along main roads in the communes.

During the intervention roll-out, two CSBs assigned to the intervention group were found not to have the technical capacity for the intervention and one did not perform pregnancy care. As the study design was built on a random assignment, these CSBs and women living in their catchment areas were included in the analysis. Two additional CSBs, that were semi-private in 2017 and became fully public only in 2019, were added to the intervention group to address the reduced number of CSBs that implemented the intervention. These two additional CSBs were excluded from analysis due to their non-random assignment.

### Data collection

Several data sources were used for the process evaluation: a quantitative survey of postpartum women, a survey of healthcare providers, and qualitative interviews with women, healthcare providers, and implementation team members. We also reviewed implementation protocols completed by implementing agents for any relevant information.

For the quantitative population-based survey among postpartum women (March – December 2022), we randomly selected up to four census enumeration areas (Fokontany) in each CSB catchment area. The research team trained local individuals who were familiar with the local dialect and context and experienced with data collection for four days to screen eligibility and conduct computer-assisted personal interviews using REDCap. These data collectors visited every second door in the selected Fokontany to identify eligible women for the interview, i.e. those who completed their pregnancy between July 2, 2020, and December 31, 2021, were at least 18 years old, and provided verbal consent. The interviews covered socioeconomic characteristics, pregnancy, birth, expenses, saving behavior, decision-making, post-partum depression, and MMHW use. Questions on MMHW use were used to assess implementation fidelity and reach. If an eligible woman existed but was absent in three attempts of interviewing her, a short interview covering socioeconomic characteristics was conducted with any available household member to compare the survey sample with eligible but absent women.

The healthcare provider survey (August – September 2022) was conducted by members of the research team. It covered all CSBs and referral hospitals in the trial, collecting information on the number of ANC visits, births, complications, medication distributed, number of staff, medical expenses based on responses of facility staff and provider records from 2020 and 2021. In intervention facilities, the person in charge of the MMHW tool was asked about their perception of the tool. Responses to these latter questions informed the assessment of implementation fidelity as well as barriers and facilitators.

Qualitative data was collected through in-depth, in-person interviews with 42 women and 4 family members in catchment areas of intervention CSBs, 23 health facility staff of intervention CSBs, and 3 implementation team members (August – November 2022). These interviews were conducted by two research assistants with previous experience in qualitative data collection who were hired specifically for this purpose, were fluent in Malagasy and French, and who underwent a two-day training and four half-day refreshers. To obtain a diversity of perspectives, purposive sampling was used. Data collectors were trained by the research team in conducting the interviews with semi-structured interview guides and regular debriefings between data collectors and the research team ensured ongoing refinement of these guides to collect rich information. Interviews were transcribed verbatim by the data collectors and translated from Malagasy into English by professional translators. While the original purpose of the qualitative data collection was not the process evaluation, the data contained relevant information for this study. Topics covered in interviews with eligible women and family members included the decision to register (or not) as well as MMHW usage. Interviews with health facility staff and implementation team members especially covered the perception of the tool, its implementation in health facilities, and perceived sustainability.

### Data analysis

The process evaluation started with the analysis of quantitative data, while qualitative findings were integrated to guide the interpretation of quantitative findings.

We quantitatively assessed the fidelity and reach of the intervention using five binary implementation indicators: whether a woman (a) had heard of the MMHW (*heard of)*, (b) could explain what the MMHW is (*knew*), (c) had registered with the MMHW (*registered*), (d) had used the MMHW to save (*saved*), (e) had used the MMHW to pay for costs of childbirth *(used)*. We used descriptive statistics and two-sided t-tests to compare characteristics of women exposed and unexposed to the intervention, i.e. living in catchment areas of intervention and control CSBs. Similarly, we used two-sided t-tests to compare characteristics of women and perceptions of health facility staff between facilities in the intervention group with high and low implementation fidelity. We excluded women in catchment areas of the two CSBs that were added to the intervention group after randomization.

To investigate barriers and facilitators to implementation and understand variation in take-up, we quantitatively compared CSBs in the intervention group by level of implementation quality. For this purpose, we retrospectively defined CSBs which were more successful in informing women and had a higher share of women in their catchment area who had heard of the MMHW, i.e. larger than the 25th percentile in the intervention group (30%), as CSBs with high information quality. CSBs with a share lower than the 25th percentile were defined as having low information quality. Similarly, we defined as usage quality how successful CSBs were in encouraging use of the MMHW, which goes beyond informing women about the tool and may be affected by different barriers. Intervention CSBs with a higher share of women in their catchment area who used the MMHW out of those who had heard of it, i.e. higher than the 25th percentile (16%), were defined as those with high usage quality, as opposed to those with low usage quality, i.e. a share lower than the 25th percentile.

The analysis of the qualitative transcripts was guided by Reflexive Thematic Analysis [18]. Respective codebooks for each respondent group were developed by three researchers and continuously refined among the research team. Qualitative information that related to the quantitative analysis of this study was integrated to complement quantitative results.

## Results

### Sample description of women respondents

Amongst the CSB control and intervention catchment areas, 6,323 women were identified as eligible for participation. Of these, 6,243 women (98.7%) consented and completed the full quantitative interview, while 300 household members participated in a shorter interview about absent eligible women.

Most women surveyed were under 35 years, with 43% aged 17 - 24 years, and 44% aged 25 - 34 years. 37% had one child, 32% had two, and 91% were married (see Table 1 for a summary of socioeconomic characteristics). Some women reported having no children due to child loss, as eligibility was based on the timing of the most recent pregnancy rather than childbirth. Regarding education, 71% completed secondary education or higher. Nearly half (45%) were unemployed, and 51% reported receiving no regular income, likely due to having very young children. The overwhelming majority of women (90%) lacked health insurance. Access to a mobile phone, which was needed to engage with the intervention, was reported by 58% and 22% of those stated that the phone was not their own. Reported characteristics of absent women differed slightly from the characteristics of women who completed the full interview (see Table 1). They were less likely to be under 25 years old and more likely to be between 25 and 34 years old. Absent women were also more likely to have access to mobile phones as well as personal assets like televisions, motorcycles, or cars, suggesting they were wealthier and more likely to be employed, possibly due to being older and having experienced previous births.

**Table 1:** Sample characteristics of individual-level survey

		Full interviews				Absent Sample	
Characteristic	N	Mean (All)	Mean (Control)	Mean (Intervention)	p-value	Mean	p-value
**Age of woman**	6243						
17–24 years		0.4282	0.4352	0.4220	0.2936	0.3244	0.0004
25–34 years		0.4391	0.4425	0.4361	0.6111	0.5251	0.0034
35–55 years		0.1328	0.1223	0.1419	0.0229	0.1505	0.3792
**Married**	6242	0.9100	0.9150	0.9056	0.1939		
**Number of children**	6235						
0 child		0.0027	0.0024	0.0030	0.6674		
1 child		0.3660	0.3777	0.3559	0.0739		
2 children		0.3192	0.3165	0.3215	0.6746		
3 children		0.1761	0.1691	0.1821	0.1800		
4 children		0.0794	0.0792	0.0795	0.9612		
5 or more children		0.0566	0.0550	0.0580	0.6074		
**Level of education**	6240						
No education		0.0167	0.0162	0.0170	0.8043		
Primary education		0.2723	0.2763	0.2688	0.5025		
Secondary education		0.5947	0.6041	0.5865	0.1580		
Higher education		0.1163	0.1033	0.1277	0.0027		
**Receives regular income**	6230	0.4913	0.4832	0.4984	0.2340		
**Type of employment**	6242						
Wage earner		0.0572	0.0542	0.0598	0.3487		
Self employed		0.3448	0.3161	0.3696	0.0000		
Family farm/livestock/fishing		0.1440	0.1976	0.0977	0.0000		
Student		0.0046	0.0048	0.0045	0.8374		
Not working		0.4518	0.4342	0.4670	0.0094		
Other		0.0210	0.0225	0.0197	0.4525		
**Access to mobile phone**	6242	0.5809	0.5639	0.5956	0.0115	0.6800	0.0007
**Phone ownership**	3630						
Own phone		0.7793	0.7681	0.7886	0.1380		
Spouse’s phone		0.1774	0.1836	0.1723	0.3774		
Someone else’s phone		0.0433	0.0483	0.0391	0.1721		
**Used mobile money, excl. MMHW**	5152	0.4602	0.4668	0.4534	0.3332		
**Television**	6231	0.5110	0.4524	0.5616	0.0000	0.6800	0.0000
**Vehicle ownership**	6234						
No vehicle		0.7713	0.7616	0.7796	0.0913	0.7181	0.0335
Bicycle		0.1322	0.1446	0.1214	0.0069	0.1074	0.2153
Motorcycle		0.0911	0.0927	0.0897	0.6795	0.1309	0.0209
Car		0.0281	0.0291	0.0272	0.6588	0.0973	0.0000
Other vehicles		0.0074	0.0083	0.0066	0.4274	0.0101	0.5994
**Insurance**	6239						
None		0.9030	0.9053	0.9010	0.5694		
Community-based medical health insurance		0.0058	0.0045	0.0069	0.2151		
Microfinance-based scheme		0.0006	0.0003	0.0009	0.3910		
Employment-based scheme		0.0688	0.0698	0.0679	0.7631		
Association/NGO		0.0051	0.0041	0.0060	0.3123		
Other		0.0178	0.0166	0.0188	0.5030		

Note: This table shows the shares of respondents falling into the category of the respective characteristic, for the sample giving full interviews (all, control, intervention) and the sample giving interviews for women absent at the time of the visit (absent). N gives the number of observations in the full interview sample for which the information is available. The *p*-value for the full interviews gives the *p*-value of a t-test of the difference between control and intervention group. The column “p-value“ under absent interviews gives the *p*-value of a t-test of the difference between absent interview and the full interview

### Sample description of CSBs

Table 2 describes the CSBs included in the randomization, excluding referral hospitals, based on healthcare provider records of 2020. On average, CSBs had 1442 ANC visits and 228 births in 2020 and were staffed with an average of two doctors, one nurse, and two midwives. There were no systematic differences in these characteristics between intervention and control CSBs in 2020. Means were similar in values and not statistically significantly different between the two groups, indicating that randomization successfully created a balanced sample according to observable provider characteristics.

**Table 2:** CSB characteristics 2020

	Full sample		By assignment		
Characteristic	N	Mean	Control	Intervention	P-value
Number of ANC visits	57	1442	1453	1429	0.9392
Number of births	56	228	234	222	0.8897
Number of doctors	56	2	2	3	0.1326
Number of nurses	54	1	1	1	0.3047
Number of midwives	60	2	2	2	0.8546
Maternal mortality	39	0.0028	0.0020	0.0035	0.4319
Newborn mortality	38	0.0003	0.0007	0.0000	0.2981

Note: This table shows characteristics of CSBs included in randomization, excluding referral hospitals, based on healthcare provider data of 2020 records. N differs because data was not available for all CSBs for all indicators. The *p*-value in the last column gives the *p*-value of a t-test of the difference between control and intervention group

### Implementation fidelity and reach

Most respondents of the healthcare provider survey had heard of the MMHW, 97% in the intervention group, 88% in the control group. In the intervention group, staff knew about the MMHW from intervention workers, while the control group knew from midwives or staff of other CSBs. The initial implementation, introducing the MMHW to health facilities, therefore worked as intended.

**Fig. 1:**
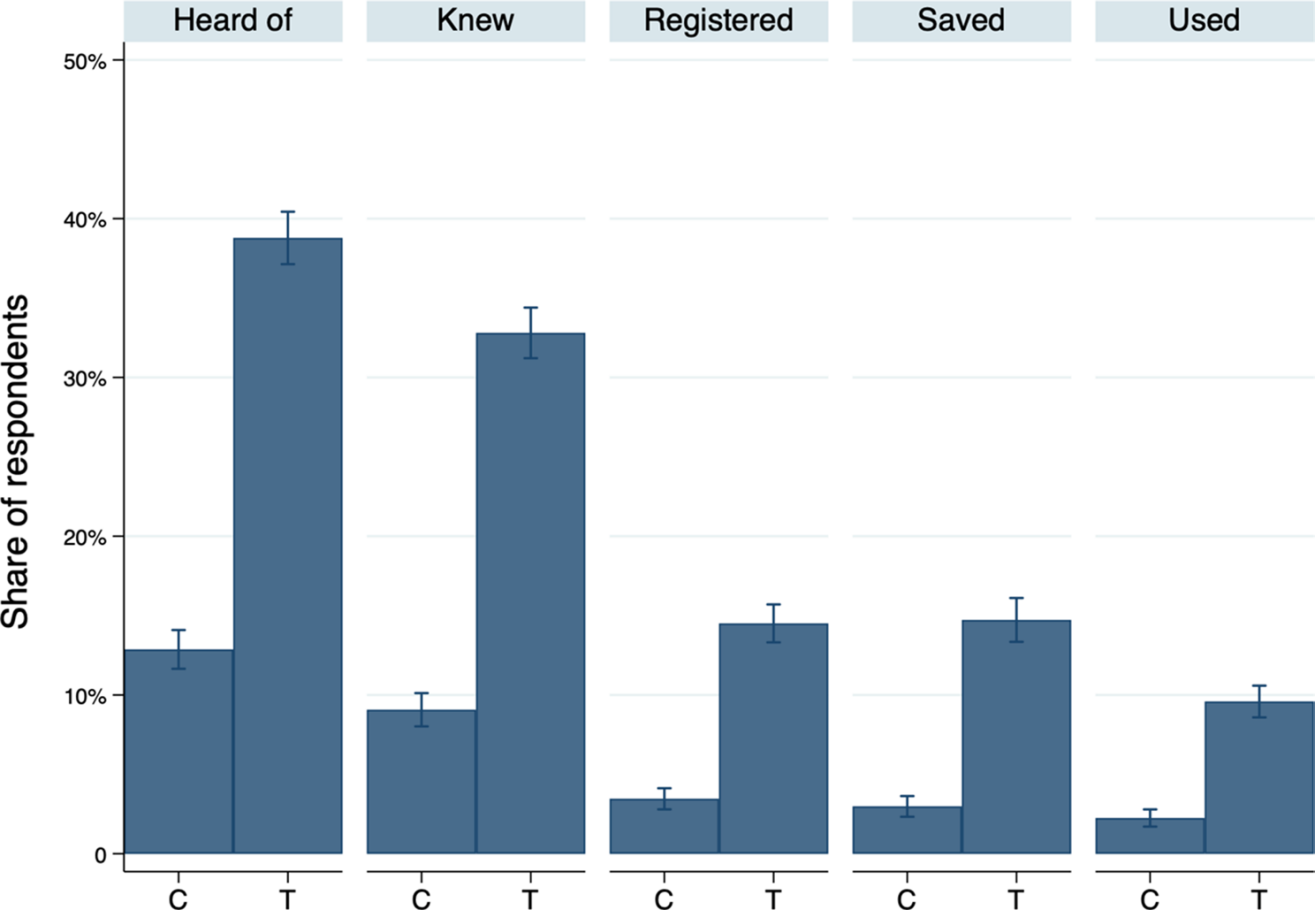
Implementation outreach. Note: share of respondents who had heard of, knew, registered with, saved with, used the MMHW, in catchment areas of control (C) and intervention (T) CSBs, with 95%-confidence intervals. *N* = 6,228. Differences between shares are statistically significant with *p*-value < 0.0001 for all five variables

The reach of the intervention in the catchment areas of CSBs can be seen in Fig. 1. Sensitization and availability of the intervention led to significantly higher take-up by women living in the catchment areas of intervention CSBs, i.e. exposed women (see Fig. 1). More than one third (38.9%) of surveyed exposed women had heard of the MMHW vs 12.9% among unexposed women. Similarly, 32.8% knew what the MMHW was, 14.5% had registered, 14.7% had saved with the MMHW and 9.6% had used it for paying, as compared to much lower shares of 9.1%, 3.5%, 3.0% and 2.3% of unexposed women. These differences are statistically significant at the 5 percent level.

**Fig. 2:**
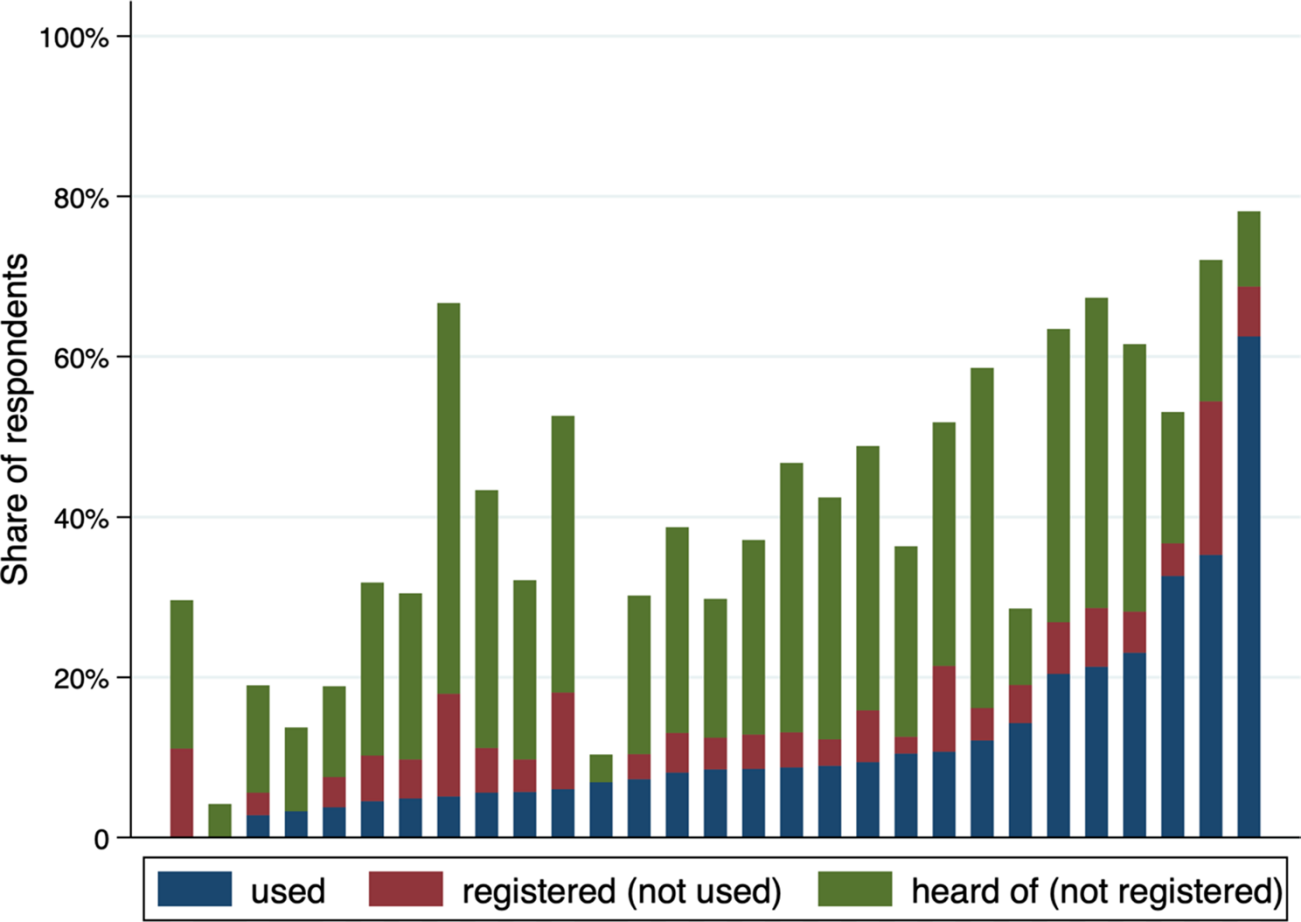
Variation in implementation outreach – intervention CSBs. Note: share of respondents who used, registered with but did not use, heard of but did not register with the MMHW, across CSBs in the intervention group. One bar per CSB. *N* = 3,338. Bars number 2, 5, and 10 are CSBs that did not actually implement the MMHW

**Fig. 3:**
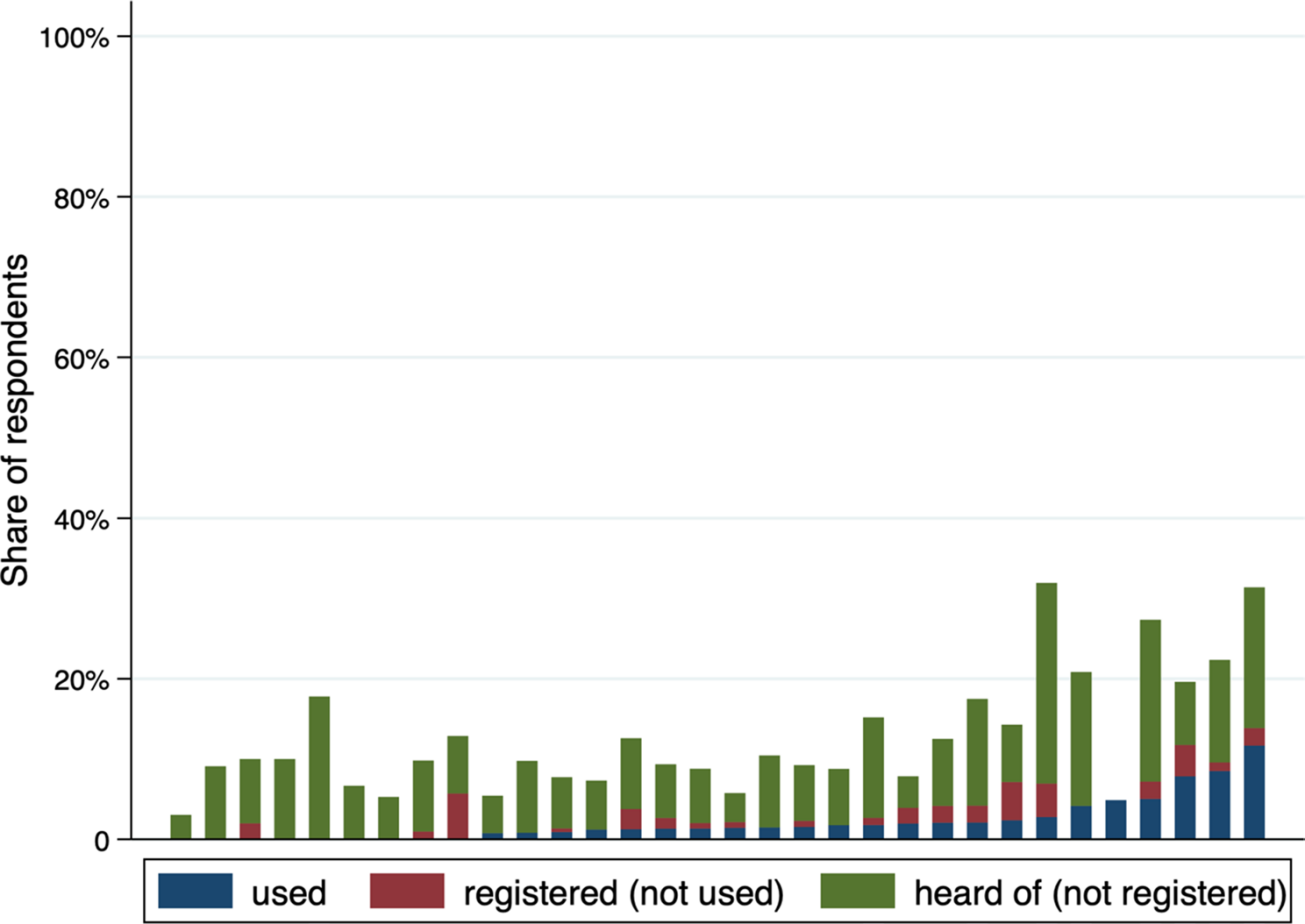
Variation in implementation outreach – control CSBs. Note: share of respondents who used, registered with but did not use, heard of but did not register with the MMHW, across CSBs in the control group. One bar per CSB. *N* = 2,890

However, these averages masked considerable variation across CSBs, especially across intervention CSBs (see Figs. 2 and 3). Some intervention CSBs had lower shares of women in their catchment area who had heard of the MMHW than in some control CSBs. The share of women who had heard of the MMHW was less than 20% for five intervention CSBs, but five control CSBs had a share of women who had heard of the MMHW of more than 20%. Moreover, the share of women who registered with or used the MMHW out of those who had heard of the tool varied greatly across CSBs. In one intervention CSB, 88% of those who had heard of the MMHW registered with the tool and in four others, the share was above 60%, while in eight other intervention CSBs, the share was below 30%.

### Barriers and facilitators to implementation: facility characteristics and implementation processes

We assessed whether observable CSB characteristics according to provider records in 2020 differed by implementation quality (see Table 3). These observable characteristics were unlikely affected by the intervention in 2020. CSBs with high information quality seemed slightly larger, with more ANC visits, higher number of births, and more doctors than those with low information quality. However, these differences were not statistically significant, likely also due to the small sample size. Similarly, CSBs with high usage quality had more ANC visits, births, and doctors (again not statistically significant) than CSBs with low usage quality. While these observable characteristics suggest that larger CSBs had better implementation quality, they do not provide an explanation for this difference.

**Table 3:** CSB characteristics 2020 by implementation quality

			By share of women who heard of MMHW (information quality)			By share of women who used MMHW out of those who had heard (usage quality)		
Characteristic	N	Mean	Low	High	P-Value	Low	High	P-value
Number of ANC visits	27	1429	1374	1557	0.5815	1402	1473	0.8269
Number of births	26	222	206	268	0.5143	199	254	0.5481
Number of doctors	27	3	2	3	0.0928	2	3	0.0670
Number of nurses	26	1	1	1	0.1438	1	1	0.4374
Number of midwives	29	2	2	3	0.4503	2	2	0.5172
Maternal mortality	21	0.0035	0.0017	0.0041	0.2173	0.0042	0.0018	0.2356
Newborn mortality	20	0.0000	0.0006	0.0000	0.3755	0.0008	0.0000	0.2462

Note: This table shows characteristics of CSBs assigned to the intervention group, excluding referral hospitals, based on healthcare provider data of 2020 records. The split into low/high share is based on the 25th percentile of the share of women who heard of/who used the MMHW in the intervention group. N differs because data was not available for all CSBs for all indicators. The columns with *p*-values give the *p*-value of a t-test of the difference by implementation quality group

According to implementation protocols and insights from implementing agents, initial problems occurred at the beginning of the intervention: SIM cards were inaccessible and registration periods too long, so that services related to prenatal consultation or maternity were not covered at the appropriate time and women had to pay out-of-pocket for these services. Furthermore, the absence of cash points in rural areas, as well as persistent mobile network problems, also caused by power cuts, affected the project repeatedly. Implementation team members reported that they attempted to address these, as well as other technical issues, through continuous contact with, and presence in, health facilities:

For network issues, there is no other solution than to wait for its return. We made arrangements with the pharmacists at CSBs: We gave [the pregnant women] the medicine while she was still there, and she would come back to pay later. Also, for those who had surgery at [a specific hospital], the patient took the medicine needed for the surgery and paid for it later, when everything was over. (implementation team member, responsible for rather rural area)

The implementation of the intervention was also impacted by the COVID-19 pandemic. COVID-19 vaccination campaigns repeatedly occupied healthcare providers, disrupting sensitization activities. Further interruptions were caused by lack or changes of staff at CSBs.

Implementation team members also highlighted the role of community health workers, noting that they were most familiar with women in their respective area and could identify those who should be targeted. However, challenges in collaboration with community health workers were also reported, especially concerning performance-based bonuses. Solutions included selective cooperation with motivated community health workers and sufficient remuneration based on objective criteria, in this case the number of enrolled women, as tracked through mobile network data on the opening of MMHW accounts.

### Barriers and facilitators to implementation: perception of MMHW among CSB staff

Differences in the perception of the MMHW among the CSB staff could have contributed to differences in implementation quality. In the healthcare provider survey, CSB staff in charge of the MMHW received questions about their perception of and satisfaction with the tool. Main reasons for staff to accept the MMHW were expected benefits for women (reported in 76% of intervention CSBs), possibility to pay through the MMHW and reduced cash payments (40%), and vouchers (40%). Among intervention CSBs, these reasons did not differ statistically significantly by implementation quality (see Table 4). Benefits for women were reported as a reason for accepting the MMHW by all CSBs in the low information quality group and by 68% of those in the high information quality group (*p*-value = 0.1241). Payments through the MMHW were reported as a reason for accepting the MMHW by 33% and 42% of CSBs, respectively (*p*-value = 0.7164). No respondent in the low information quality group but 16% in the high information quality group reported better access to care as a reason (*p*-value = 0.3196). A large majority of respondents (83%) reported that one advantage experienced when using the MMHW tool were that more women came to the CSB (100% (low) vs 78% (high), *p*-value = 0.2232). Another advantage mentioned by 25% of respondents was the secure payment (17% (low) vs 28% (high), *p*-value = 0.6052). Frequently mentioned problems were lack of connectivity (64%, 67% (low) vs 63% (high), *p*-value = 0.8822) and late payment from MMHW to the health facility (52%, 33% (low) vs 58% (high), *p*-value = 0.3138). 75% of respondents claimed that using the MMHW increased their workload (80% (low) vs 74% (high), *p*-value = 0.7834). Despite the difficulties and dissatisfaction, 79% of respondents in the intervention group stated that they would recommend the tool to other health facilities (100% (low) vs 71% (high), *p*-value = 0.1986) and all except one CSB stated that they would like the tool to continue to work at their facility. Also, 92% claimed that they encouraged pregnant women to sign up for the tool (100% (low) vs 89% (high), *p*-value = 0.4159).

**Table 4:** Perception of staff in intervention CSBs by implementation quality

			By share of women who heard of MMHW (information quality)			By share of women who used MMHW out of those who had heard (usage quality)		
Indicator	N	Mean	Low	High	P-Value	Low	High	P-value
Heard of the MMHW	29	0.9655	0.8750	1.0000	0.1062	0.8750	1.0000	0.1062
**Reasons for accepting**								
Payment through MMHW, less cash	25	0.4000	0.3333	0.4211	0.7164	0.2857	0.4444	0.4878
Training	25	0.0400	0.0000	0.0526	0.5851	0.0000	0.0556	0.5443
Ambulance	25	0.2400	0.1667	0.2632	0.6464	0.2857	0.2222	0.7512
Vouchers	25	0.4000	0.3333	0.4211	0.7164	0.5714	0.3333	0.2947
Financial benefit for CSB	25	0.1200	0.1667	0.1053	0.7014	0.0000	0.1667	0.2681
Better access to/quality of care	25	0.1200	0.0000	0.1579	0.3196	0.1429	0.1111	0.8351
Benefits for women	25	0.7600	1.0000	0.6842	0.1241	0.7143	0.7778	0.7512
Did not know they had a choice	25	0.0400	0.0000	0.0526	0.5851	0.0000	0.0556	0.5443
**Person in charge**								
Midwife	24	0.3750	0.3333	0.3889	0.8176	0.4286	0.3529	0.7416
Pharmacist	24	0.5000	0.5000	0.5000	1.0000	0.5714	0.4706	0.6701
**Main advantage**	24	0.2500						
Secure payment	24	0.8333	0.1667	0.2778	0.6052	0.0000	0.3529	0.0747
More women come to the CSB	24	0.1250	1.0000	0.7778	0.2232	0.7143	0.8824	0.3366
Midwife bonus	24	0.2917	0.0000	0.1667	0.3057	0.0000	0.1765	0.2535
Other	24	0.0417	0.1667	0.3333	0.4587	0.4286	0.2353	0.3655
Don’t know	24	0.0833	0.0000	0.0556	0.5753	0.0000	0.0588	0.5331
Refuse to answer	24	0.3750	0.0000	0.1111	0.4159	0.2857	0.0000	0.0205
**Main problem**								
Connectivity	25	0.6400	0.6667	0.6316	0.8822	0.5714	0.6667	0.6719
Lost SIM card	25	0.2400	0.3333	0.2105	0.5587	0.4286	0.1667	0.1828
Money comes late to CSB	25	0.5200	0.3333	0.5789	0.3138	0.2857	0.6111	0.1560
Errors during payment process	25	0.0400	0.0000	0.0526	0.5851	0.1429	0.0000	0.1102
Difficulty in understanding process	25	0.0400	0.0000	0.0526	0.5851	0.1429	0.0000	0.1102
Other	25	0.5600	0.3333	0.6316	0.2156	0.4286	0.6111	0.4303
**MMHW increased workload**	24	0.7500	0.8000	0.7368	0.7834	0.8571	0.7059	0.4587
**Encouraged women to sign up**	24	0.9167	1.0000	0.8889	0.4159	0.8571	0.9412	0.5196
**Would recommend MMHW to other facilities**	19	0.7895	1.0000	0.7143	0.1986	1.0000	0.7333	0.2698
**Which part of MMHW should continue?**								
Ambulance	24	0.3750	0.3333	0.3889	0.8176	0.1429	0.4706	0.1436
Voucher, ultrasound	24	0.4583	0.6667	0.3889	0.2558	0.4286	0.4706	0.8589
Training	24	0.0417	0.0000	0.0556	0.5753	0.0000	0.0588	0.5331
Payment through MMHW	24	0.2083	0.1667	0.2222	0.7834	0.0000	0.2941	0.1163
All of them	24	0.5417	0.3333	0.6111	0.2558	0.5714	0.5294	0.8589

Note: This table shows the perception of staff in CSBs assigned to the intervention group, excluding referral hospitals, based on the healthcare provider survey. The split into low/high share is based on the 25th percentile of the share of women who heard of/who used the MMHW in the intervention group. The columns with *p*-values give the *p*-value of a t-test of the difference by implementation quality group

Some healthcare providers also emphasized the value of the MMHW (referred to as m-Tomady) for high-risk cases who might be faced with high treatment costs, taking advantages of the 50% matching of deposits.

Yes. There was a woman who was pregnant with her fourth child. And her fourth child is twins and that is when we suggested to her to enter m-Tomady: ‘It will help you a lot because we do not know if you are going to give birth by normal way.’ And in fact she did not have a normal childbirth. It helped her a lot because she had to have a surgery. The SIM card helped her a lot financially. (CSB midwife)

However, healthcare staff also highlighted some practical concerns they faced. They found that payment processes were too lengthy for involved staff and that it was relatively difficult to solve problems.

As soon as we send the invoice today, if you can, send it today. Because for us, when it comes to payment, immediately you go and make the payment and there should not be even one ariary missing. Sometimes I have to find a way, because if I have 1,000 ariary missing from the CSB’s money, they will all come down here to see that Madame [name] has embezzled. (CSB pharmacist)

While staff was used to processing paper bills and could solve problems arising in their usual process, they had to wait for MMHW staff to solve problems if any arose with this tool. This affected their routine activities around prenatal consultations as patients had to wait longer than usual.

What is difficult about using m-Tomady [the MMHW software] is the network. The network is bad, there are times when I receive someone, it is blocked, you make the person wait. But here, you do not keep people waiting for long. There are also times when there is no phone credit that is what I found difficult and painful. And the person is waiting! We cannot keep people waiting for long! (CSB staff)

Implementation protocols mentioned many instances of complaints about delayed payments and reimbursements as well as technical difficulties with the platform and tablets used in all CSBs, as late in the project as April and May 2022. These practical barriers had direct impact on take-up. According to qualitative interviews, some midwives forced patients to pay with cash instead of using savings in the wallets, for fear that reimbursements would arrive too late for them to stock up on medicines.

Employees are fed up when they suffer to get their money. And in the hospital there are those who tell pregnant women directly: ‘No, m-Tomady does not work here’. (CSB pharmacist)

Dissatisfaction with payment processes and commissions also disrupted sensitization activities at some CSBs. Qualitative interviews revealed that one midwife explicitly discouraged women from registration because it would lead to cuts in her own payment. Being confronted with technical problems, implementation team members valued the ongoing liaison with the technical and financial teams, that supported the resolution of issues, either via phone or direct presence.

At the beginning there was one person who was particularly involved in the payments; they shared the office with us. The person doesn’t necessarily have to be in the same office, but there should be a special person specifically for this project, because the financial matters are very delicate in CSBs and the government offices. (implementation team member, project coordination)

### Barriers and facilitators to implementation: women’s characteristics

We further examined whether women residing in catchment areas of intervention CSBs with low and high implementation quality differed in sociodemographic characteristics (see Table 5). No systematic differences were found in age, marital status, number of children, education, employment status, phone ownership, asset ownership. However, more women in catchment areas of CSBs with high usage quality than in those with low usage quality lacked health insurance (91% (high) vs 87% (low), *p*-value = 0.0002). Further differences emerged regarding the choice of location for childbirth and ANC visits. In catchment areas of CSBs with low information and usage quality, women were more likely to deliver in private clinics and less likely to deliver in the CSB of their Fokontany. While women were equally likely to have gone for any ANC visit, women in catchment areas of CSBs with low information quality were less likely to use the CSB of their Fokontany and more likely to use private or faith-based clinics or hospitals for ANC. These associations do not imply causality. Women may have avoided the CSB of their Fokontany because of factors linked to low implementation quality, including lack of encouragement to use the MMHW at the facility, restricted access to CSB services, or they may have preferred private clinics for unrelated reasons and therefore did not hear about the MMHW. Such an unrelated reason, the particular timing of birth, is illustrated by the experience of one woman:

I gave birth at home but despite that I returned to the CSB II […] I gave birth at night and that is why I gave birth at home. The doctor at the CSB II also told me to go and give birth with this midwife if it would happen at night. (mother who registered with the MMHW)

**Table 5:** Sociodemographic characteristics of women in catchment areas of intervention CSBs by implementation quality

			By share of women who heard of MMHW (information quality)			By share of women who used MMHW out of those who had heard (usage quality)		
Characteristic	N	Mean	Low	High	P-Value	Low	High	P-value
**Age of woman**	3514							
17–24 years		0.4180	0.4238	0.4156	0.6544	0.4345	0.4122	0.2392
25–34 years		0.4408	0.4343	0.4435	0.6151	0.4236	0.4469	0.2207
35–55 years		0.1411	0.1419	0.1408	0.9340	0.1419	0.1409	0.9369
**Married**	3347	0.9056	0.9115	0.9031	0.4496	0.9103	0.9039	0.5751
**Number of children**	3344							
0 child		0.0030	0.0020	0.0034	0.5017	0.0068	0.0016	0.0154
1 child		0.3559	0.3414	0.3620	0.2561	0.3614	0.3539	0.6913
2 children		0.3215	0.3192	0.3224	0.8572	0.3034	0.3279	0.1815
3 children		0.1821	0.1984	0.1752	0.1132	0.1955	0.1774	0.2325
4 children		0.0795	0.0796	0.0795	0.9987	0.0727	0.0820	0.3840
5 or more children		0.0580	0.0594	0.0574	0.8218	0.0602	0.0572	0.7437
**Level of education**	3345							
No education		0.0170	0.0251	0.0136	0.0187	0.0159	0.0174	0.7627
Primary education		0.2688	0.2533	0.2753	0.1885	0.2557	0.2734	0.3082
Secondary education		0.5865	0.5819	0.5885	0.7231	0.5841	0.5874	0.8632
Higher education		0.1277	0.1397	0.1226	0.1744	0.1443	0.1217	0.0844
**Receives regular income**	3337	0.4984	0.4960	0.4994	0.8579	0.5194	0.4908	0.1470
**Type of employment**	3347							
Wage earner		3347	0.0598	0.0583	0.0604	0.8163	0.0590	0.0600
Self employed		3347	0.3696	0.3568	0.3750	0.3185	0.3655	0.3710
Family farm/livestock/fishing		3347	0.0977	0.0905	0.1008	0.3585	0.0851	0.1022
Student		3347	0.0045	0.0060	0.0038	0.3832	0.0045	0.0045
Not working		3347	0.4670	0.4955	0.4549	0.0316	0.4869	0.4599
Other		3347	0.0197	0.0121	0.0230	0.0382	0.0170	0.0207
**Access to mobile phone**	3514	0.5962	0.6213	0.5856	0.0489	0.6046	0.5932	0.5466
**Phone ownership**	1996							
Own phone		0.7886	0.7785	0.7931	0.4627	0.7926	0.7871	0.7892
Spouse’s phone		0.1723	0.1710	0.1729	0.9162	0.1815	0.1690	0.5106
Someone else’s phone		0.0391	0.0505	0.0340	0.0796	0.0259	0.0440	0.0649
**Used mobile money, excl. MMHW**	2543	0.4534	0.4762	0.4417	0.0987	0.4244	0.4642	0.0738
**Television**	3510	0.5655	0.5973	0.5521	0.0135	0.5527	0.5701	0.3592
**Vehicle ownership**	3508							
No vehicle		0.7791	0.7793	0.7790	0.9856	0.7690	0.7827	0.3896
Bicycle		0.1203	0.1142	0.1229	0.4710	0.1334	0.1156	0.1542
Motorcycle		0.0904	0.0960	0.0880	0.4518	0.0954	0.0886	0.5311
Car		0.0291	0.0326	0.0276	0.4157	0.0260	0.0302	0.5216
Other vehicles		0.0068	0.0077	0.0065	0.6963	0.0098	0.0058	0.2104
**Insurance**	3345							
None		0.9010	0.8974	0.9026	0.6448	0.8693	0.9124	0.0002
Community-based medical health insurance		0.0069	0.0070	0.0068	0.9397	0.0125	0.0049	0.0187
Microfinance-based scheme		0.0009	0.0010	0.0009	0.8909	0.0000	0.0012	0.3006
Employment-based scheme		0.0679	0.0744	0.0651	0.3250	0.0841	0.0621	0.0258
Association/NGO		0.0060	0.0060	0.0060	0.9778	0.0068	0.0057	0.7069
Other		0.0188	0.0171	0.0196	0.6321	0.0295	0.0150	0.0065
**Birthplace**	3331							
Home		0.1693	0.1806	0.1645	0.2576	0.1682	0.1697	0.9177
Matron’s home		0.0183	0.0303	0.0132	0.0008	0.0160	0.0191	0.5560
CSB of Fokontany		0.1702	0.0444	0.2235	0.0000	0.1476	0.1783	0.0383
Other CSB		0.1780	0.1736	0.1799	0.6614	0.1419	0.1909	0.0011
Referral hospital		0.1237	0.1292	0.1214	0.5323	0.1384	0.1184	0.1229
Private clinic		0.2270	0.3148	0.1897	0.0000	0.2700	0.2116	0.0004
Private hospital		0.0742	0.0858	0.0692	0.0958	0.0709	0.0753	0.6730
Faith-based clinic or hospital		0.0069	0.0061	0.0073	0.6998	0.0069	0.0069	0.9868
Other		0.0324	0.0353	0.0312	0.5394	0.0400	0.0297	0.1386
**Any ANC visit**	3348	0.9806	0.9779	0.9817	0.4624	0.9773	0.9818	0.4103
**Location of ANC visit**	3283							
Own or matron’s home		0.0079	0.0103	0.0069	0.3227	0.0093	0.0074	0.5971
Any CSB		0.6424	0.5324	0.6887	0.0000	0.6283	0.6474	0.3164
CSB of Fokontany		0.3201	0.1932	0.3736	0.0000	0.3624	0.3051	0.0020
Referral hospital		0.0533	0.0555	0.0524	0.7166	0.0523	0.0537	0.8744
Private or faith-based clinic or hospital		0.3220	0.4296	0.2766	0.0000	0.3415	0.3150	0.1540
Other		0.0228	0.0277	0.0208	0.2224	0.0279	0.0211	0.2503
**Source of information about MMHW**	1297							
Family or friends		0.1126	0.1023	0.1146	0.6032	0.1649	0.0982	0.0018
Midwife		0.6191	0.6140	0.6201	0.8645	0.4982	0.6523	0.0000
Doctor		0.0640	0.0651	0.0638	0.9414	0.0466	0.0688	0.1804
Posters		0.2035	0.2047	0.2033	0.9649	0.2688	0.1857	0.0022
Home visit		0.0725	0.0465	0.0776	0.1081	0.0896	0.0678	0.2132
Road show		0.0139	0.0233	0.0120	0.1984	0.0215	0.0118	0.2193
Facebook		0.0023	0.0000	0.0028	0.4399	0.0036	0.0020	0.6182

Note: This table shows the shares of respondents falling into the category of the respective characteristic, for the sample with full in the catchment area of intervention CSBs, split by implementation quality. The split into low/high share is based on the 25th percentile of the share of women who heard of/who used the MMHW in the intervention group. The columns with *p*-values give the *p*-value of a t-test of the difference by implementation quality group. N gives the number of observations for which the information is available

Women learned about the MMHW from different sources, including midwives (mentioned by 55% in the quantitative survey), posters in health facilities (18%), and family or friends (17%). Women in treatment areas were more likely to have heard about the MMHW from midwives than in control areas (63% vs 48% among those who had heard about the MMHW, *p*-value = 0.0063), but less likely from family and friends (13% vs 21%, *p*-value = 0.1234). This difference arises from the intervention design. Among intervention CSBs, the share of women who had heard of the MMHW from the respective information source did not differ by the CSBs’ information quality (see Table 5). This suggests that no source was more successful in informing about the tool. However, the share of women who had heard of the MMHW from family and friends or midwives differed by CSBs’ usage quality. In high usage quality CSBs, 65% learned from midwives, compared to 49% in low-usage quality CSBs (*p*-value = 0.0000), and 10% heard from family and friends, compared to 16% in low-usage quality CSBs (*p*-value = 0.0018). Women in high-usage quality CSBs were also less likely to hear from posters (19% (high) vs 27% (low), *p*-value = 0.0022). This indicates that midwives were more effective in promoting MMHW use. We can look at this slightly differently by measuring the correlation between having registered with the tool and having heard about it from specific information sources. A statistically significantly positive correlation was found between registration and having heard about the MMHW from midwives, and a statistically significantly negative correlation with family or friends, in both high and low quality CSBs.

Qualitative interviews with eligible women on enrollment decisions, analyzed in more detail elsewhere, confirmed the importance of the information sources [16]. These interviews also identified barriers such as concerns of fraud, technical problems, transparency issues, as well as project-related barriers, such as the need for an ID card that not all eligible women possessed [16].

Many registered women emphasized how the MMHW supported them during pregnancy and childbirth. However, several respondents confirmed problems with reimbursement, technical issues, and miscommunication at health facilities regarding payment status.

Already being registered, some women still expressed a fear of new technologies, concerns about accessibility, and worries about where savings would be stored and how they could be accessed by users of the MMHW.

She [the midwife] gave me a SIM card that couldn’t be opened. I went back to her and she told me to go to an Airtel shop. That I should go there with an ID card, and that only me could do it. So I just gave up on it. (mother who registered with MMHW)

Also, the fact that MMHW usage was limited to participating CSBs restricted use, particularly when women sought care elsewhere, e.g., due to relocation.

## Discussion

This study assessed the take-up of a mobile money-based savings tool for pregnancy, the MMHW, among pregnant women in Madagascar’s Analamanga region, revealing substantial variation across multiple stages of implementation – awareness, adoption, use. While women in intervention catchment areas were significantly more likely to have heard about, registered with, and used the MMHW compared to those in control areas on average, these rates varied greatly between CSBs. Awareness ranged from 4% to 78% across catchment areas, with further variation in registration among those aware of the tool. From an implementation science perspective, these patterns point to heterogeneity in adoption and reach, as well as differences in implementation quality across sites.

Our findings suggest that local delivery processes and contextual factors played a critical role in shaping uptake. In particular, the source of information emerged as determinant: In intervention CSBs with high usage quality, midwives were more frequently mentioned as information source about the MMHW than in CSBs with low usage quality, whereas family and friends or posters were less frequently mentioned. This finding was supported in the qualitative data [16] and highlights the importance of trusted intermediaries and provider engagement in fostering uptake. Within frameworks such as the Consolidated Framework for Implementation Research (CFIR), this can be interpreted as reflecting the influence of both “inner setting” factors (e.g., provider engagement and communication practices) and “characteristics of individuals” (e.g., trust and perceived credibility), which shape adoption [19].

We identified limited evidence on similar savings tools that is directly comparable, but existing studies can be interpreted through related implementation constructs. In a study in Malawi, mobile money accounts were opened for participating entrepreneurs who also received basic training on mobile money and were encouraged to save [20]. Almost all entrepreneurs opened an account and 73% made at least one deposit. While uptake in terms of saving rate was higher than in our study, this difference could reflect greater resource availability among entrepreneurs, a factor associated with improved feasibility and adoption. A smaller study among nomadic populations in Kenya, where individuals testing positive for brucellosis received a mobile health wallet with funds for treatment, found 88% acceptance of the wallet [21]. This intervention differed from ours in that the wallet was for acute treatment and no active saving was required from its users, thereby lowering barriers to participation.

The mobile health wallet M-TIBA in Kenya, which allows users to save funds dedicated to accessing healthcare services, underscores the role of incentives in shaping uptake [22]. M-TIBA offered incentives for its first 100,000 users to encourage sign-up, but usage declined once the bonus scheme ended [23]. The MMHW also provided incentives, including vouchers and 50% matching of deposits by a third party. Taken together, these comparisons suggest that variation in adoption across settings can be understood in terms of broader implementation constructs – particularly perceived benefits, resource constraints, and the design of incentive structures.

Several factors may explain why potential beneficiaries did not hear about or use the MMHW, operating on both the providers’ and on women’s sides and reflecting common implementation barriers. On the provider side, an earlier MMHW perception study identified the disruption of informal benefits for healthcare providers related to the cash-based payment system as potential barrier for the use of the MMHW at CSBs [24]. This points to issues of incentive alignment and compatibility with existing practices, which are central determinants of implementation success. The high transparency introduced by the MMHW may have reduced staff benefits from out-of-pocket payments, a concern supported by qualitative interviews. In the quantitative survey, while not statistically significant, staff in CSBs with low quality implementation were less likely to mention MMHW-based payment as a reason to accept the MMHW and less likely to mention secure payment as an advantage of the MMHW compared with CSBs with high quality implementation. This suggests lower perceived relative advantage and weaker buy-in among providers.

Overall, staff seemed to differ in their enthusiasm for the tool, reflecting differences in acceptability and implementation climate across facilities. Operational challenges likely contributed to perceptions of reduced feasibility. Late payments were mentioned as a problem more frequently by CSBs with high implementation quality, possibly due to higher claim volumes. As reported above, implementation protocols and qualitative interviews documented dissatisfaction with delayed payments, technical difficulties, and lengthy payment processes. These challenges are also reflected in three quarters of respondents in the healthcare provider survey mentioning an increased workload, highlighting concerns about implementation burden, a possible barrier to sustained engagement. Initial implementation challenges may have contributed to frustration among both CSB staff and potential users, potentially fueling negative rumors about the tool.

Both qualitative and quantitative evidence underscore the central role of facility staff, particularly midwives, in shaping adoption and reach. As key intermediaries, they influenced whether women became aware of and registered with the MMHW. However, if staff were not convinced of the tool’s benefits, they may have been less likely to promote its use. This reluctance can be understood in terms of both individual-level factors (e.g., perceived loss of informal benefits, increased workload) or concerns about overall service delivery (risk of medicine stock-out). Both aspects illustrate how provider buy-in is shaped by both personal incentives and perceptions of system readiness.

Other process evaluations of health services interventions identified similar barriers to implementation, including loss of financial autonomy by health facilities and unreliable disbursement of funds or benefits, perceived amount of work burden, and opportunity costs, i.e. whether participation in the intervention led to loss of benefits or profits [25–29]. Within implementation science, these factors can be conceptualised as part of the inner setting and implementation processes, including the degree of stakeholder engagement and the compatibility of an intervention with organizational practices, which seem crucial for ensuring the intervention success [27]. Our results further highlight the importance of effective coordination between health facilities and implementation, including technical and financial teams. This underscores how implementation success depends not only on intervention design but also on the strength of coordination structures and communication channels across involved actors.

At the user level, several factors may have limited adoption and engagement with the MMHW. First, the survey only captured eligible women who were present at their residence at the time of the data collection. Short interviews with household members of eligible absent women showed that absent women differed from those who participated in the survey. Studies on mobile money adoption showed that wealthier, better educated individuals, and mobile phone owners are more likely to adopt mobile money [8]. Case studies of digital financial services for health also highlighted digital illiteracy and low penetration of mobile phone use as barrier to adoption [30]. As low mobile phone ownership was identified as a potential barrier in the earlier MMHW perception study [24], mobile phone access may have been a barrier in this study, with less than 60% of women having access to a phone, compared to 80% in a design study of the MMHW [14]. It is necessary to differentiate here between ownership and access as owning a mobile phone was not a requirement to register with the MMHW. However, limited access to a phone may limit familiarity with mobile technology, thereby lowering self-efficacy and increasing perceived complexity [16], both of can inhibit adoption [31]. Possibly take-up of the tool was higher among all eligible women than in the survey sample.

Illiteracy, identified as potential barrier to adoption of mobile money-based services [32], was not a major concern in this setting where most women had secondary or higher education. Nevertheless, as reported above, qualitative interviews revealed remaining concerns among potential users about how and where savings would be stored. This points to limitations in sensitization and communication strategies, suggesting that sensitization activities may not have adequately communicated the practical functioning of the MMHW.

Finally, the COVID-19 pandemic represents an important external contextual factor influencing implementation. While the pandemic severely impacted Madagascar’s healthcare system [33], it also impacted the implementation of the intervention and likely impacted take-up. In addition to vaccination campaigns disrupting sensitization activities, changes in country-specific regulations and recommendations about personal movements as well as individual beliefs about infection risks likely influenced women’s decision to visit CSBs for ANC and birth. Qualitative case studies from Kenya and Rwanda further suggest that the COVID-19 pandemic reduced people’s ability to save for health [30]. These disruptions can be understood as part of the broader outer setting, affecting both the delivery of the intervention and users’ capacity to engage with it. Overall, the COVID-19 pandemic was likely one of the reasons lowering registration and use of the MMHW.

Taken together, these findings could speak to broader dynamics of implementing digital financial health interventions in low-resource settings. The observed variation in awareness, registration, and use highlights that adoption is not solely a function of intervention design, but is critically shaped by how the intervention is introduced and communicated at the local level. Variation in implementation quality emerges from the interaction of provider incentives, organizational capacity, user characteristics, and external shocks – highlighting the inherently multi-level nature of implementation processes.

### Strengths and limitations

This study evaluates the implementation fidelity of an intervention introducing a mobile money-based savings tool using multiple data sources, enriching quantitative results with qualitative data. It compares high and low performing facilities and estimates associations between individual- and provider-level characteristics and implementation quality, with the goal to understand barriers and facilitators of implementation. The study’s main limitation is a lack of detail in implementation protocols. While the protocols mention providers’ concerns and specific cases or discussions between implementers and healthcare providers, they do not systematically document which provider faced which problem. They also do not capture accurately the timing, place, and number of sensitization events. As a result, it is impossible to assess whether certain events or their frequency are associated with implementation quality. Given that the qualitative components of the trial were conceptualized for different purposes than this process evaluation study, we also cannot retrospectively assign statements or findings by implementation quality. Moreover, the qualitative interviews were carried out by data collectors hired by the implementing organization and respondents may have responded more favorably toward the intervention due to social desirability bias.

One concern regarding the overall design of the MMHW concerns its sustainability. The MMHW included project-funded incentives such as deposit matching, vouchers, and per-patient payments to healthcare staff. These incentives would not automatically continue beyond the trial. However, the intervention was developed in close collaboration with the Malagasy Ministry of Health and aligned with the government’s strategic UHC agenda. The implementing organization also coordinated with the World Bank, which has supported Madagascar’s health financing reforms through the Global Financing Facility and related programs, situating the MMHW within a broader ecosystem of health financing reform. The core mechanism of the intervention was not designed as a permanently subsidized service but as an approach that could be adopted by government as part of a broader strategy to reduce financial barriers to maternal healthcare. Nevertheless, the experience [23] in Kenya, where usage declined after incentive schemes ended, underscores that sustaining user engagement beyond an initial incentive phase remains a challenge for digital health financing tools.

## Conclusion

This study highlights the complexity of introducing a mobile money-based savings tool for obstetric care and the importance of conducting implementation research to open the “black box” of complex health system interventions. The sensitization strategies were effective in generating higher take-up in intervention than control areas, but there was considerable variation in take-up. In this study, healthcare providers were involved as users of the tool and in encouraging their patients to register, making them crucial for intervention take-up. Their perception of financial disadvantages and risks, i.e. less informal payments and payment delays compared to immediate cash payments by patients, were mentioned as barriers. Future interventions should focus on meeting the needs of both beneficiaries and providers. Creating trust and transparent benefits for all stakeholders seems crucial to scaling and sustaining mobile health savings tools. Adaptive implementation strategies to continuously resolve problems are needed to realize the potential of digital health financing tools to contribute towards achieving universal health coverage.

## Data Availability

The quantitative datasets used and/or analyzed during the current study are available in the Göttingen Research Online repository: 10.25625/KWT5KG. Qualitative data are available upon reasonable request.

## Abbreviations

4MOTHERS trial: Madagascar Mobile Money for Maternal Healthcare-Related Spending trial
ANC: Antenatal care
CSB: Centre de Santé de Base
MGA: Malagasy Ariary
MMHW: Mobile Maternal Health Wallet
USD: United States Dollar

## Footnotes

1 According to OANDA currency converter rate for January 1^st^, 2021.

2 At the beginning of the intervention, the bonus was provided per claim filed. After January 2021, the bonus was provided per patient and month with a claim sent via the MMHW platform.
